# Conference Report: Application Portability Profile and Open System Environment Users’ Forum Gaithersburg, MD May 11–12, 1994

**DOI:** 10.6028/jres.100.010

**Published:** 1995

**Authors:** Joseph Hungate

**Affiliations:** Systems and Software Technology Division, National Institute of Standards and Technology, Gaithersburg, MD 20899-0001

## 1. Introduction

The National Institute of Standards and Technology, Systems and Software Division, sponsored a Users’ Forum on the Application Portability Profile (APP) and Open System Environment (OSE) at NIST on May 11 and 12, 1994. This forum was the thirteenth in a continuing semiannual series on the NIST APP and its application to OSE. The APP Users’ Forums are designed to provide users and providers with the opportunity to exchange information and respond to NIST proposals regarding the evaluation and adoption of an integrated set of standards to support the APP and OSE.

A tutorial for beginners with little or no experience with the APP and OSE was held on the morning of the first day. The tutorial presented basic OSE concepts and the reference model. This Users’ Forum featured two mini-workshops: Distributed System Management and OSE Users’ Perspective. Extensive discussion took place concerning participants’ case studies, current activities, plans and lessons learned in relation to the APP/OSE in these areas. The usual presentation of standards and activities in the APP, OSE, Institute of Electrical and Electronics Engineers (IEEE), and Joint Technical Committee 1 (JTC1-international activities) was made. The next APP/OSE Users’ Forum will be held November 15 and 16, 1994, at NIST.

The APP/OSE Users’ Forum has been developed to assist federal agencies with information technology (IT) issues. Central to this assistance is publication and maintenance of a technical guidance document, the Application Portability Profile (APP), facilitating the migration to open systems. An Open System Environment encompasses the functionality needed to provide interoperability, portability, and scalability of computerized applications across networks of heterogeneous, multi-vendor hardware/software/communications platforms. The APP integrates industry, federal, national, international, and other specifications into a Federal application profile to provide the functionality necessary to accommodate a broad range of Federal information technology requirements. The Application Portability Profile (APP), The U.S. Government’s Open System Environment Profile OSE/1 Version 2.0 provides recommendations on a variety of specifications that will generally fit the requirements of U.S. Government systems. A specific organization will not necessarily require all of the recommended specifications in the APP. As the U.S. Government’s OSE profile, this guidance is provided to assist federal agencies in making informed choices regarding the selection and use of OSE specifications, and in the development of more selective application profiles based on the APP. It is directed toward managers and project leaders who have the responsibilities of acquiring, developing, and maintaining information systems supported by heterogeneous application platform environments.

## 2. Tutorial for Novices

The forum began with an introductory tutorial on the Open System Environment (OSE). The OSE forms an extensible framework that allows services, interfaces, protocols, and supporting data formats to be defined in terms of nonproprietary specifications that evolve through open (public), consensus-based forums. A selected suite of specifications that defines these interfaces, services, protocols, and data formats for a particular class or domain of applications is called a profile. Fritz Schulz presented OSE general concepts and the reference model. Gary Fisher presented the APP and the profiling process and selection.

### 2.1 OSE Reference Model

**Fig. 1:**
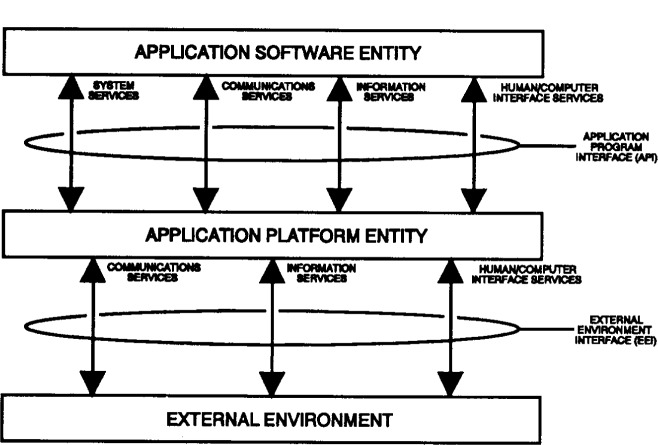
Open system environment reference model.

The Institute of Electrical and Electronics Engineers (IEEE) POSIX Working Group P1003.0 describes an OSE Reference Model (OSE/RM) that is closely aligned with the APP and that provides a framework for describing open system concepts and defining a lexicon of terms that can be agreed upon generally by all interested parties. Figure 1 illustrates the OSE/RM.

Two types of elements are used in the model: entities consisting of the application software, application platform, and platform external environment; and interfaces including the application program interface and external environment interface.

The three classes of OSE reference model entities are described as follows:

- Application Software—Within the context of the OSE Reference Model, the application software includes data, documentation, and training, as well as programs.
- Application Platform—The application platform is composed of the collection of hardware and software components that provide the system services used by application software.
- Platform External Environment—The platform external environment consists of those system elements that are external to the application software and the application platform (e.g., services provided by other platforms or peripheral devices).

There are two classes of interfaces in the OSE reference model, as described in the following paragraphs:

- Application Program Interface (API)—The API is the interface between the application software and the application platform. Its primary function is to support portability of application software. An API is categorized in accordance with the types of service accessible via that API. There are four types of API services in the OSE/RM:
  1. Human/computer interface services
  2. Information interchange services
  3. Communication services
  4. Internal system services
- External Environment Interface (EEI)—The EEI is the interface that supports information transfer between the application platform and the external environment, and between applications executing on the same platform. Consisting chiefly of protocols and supporting data formats, the EEI supports interoperability to a large extent. An EEI is categorized in accordance with the type of information transfer services provided. There are three types of information transfer services. These are transfer services to and from:
  1. Human users
  2. External data stores
  3. Other application platforms

In its simplest form, the OSE/RM illustrates a straightforward user-supplier relationship: the application software is the user of services and the application platform/external environment entities are the suppliers. The API and EEI define the services that are provided.

A profile consists of a selected list of standards and other specifications that define a complement of services made available to applications in a specific domain. Examples of domains might include a workstation environment, an embedded process control environment, a distributed environment, a transaction processing environment, or an office automation environment, to name a few. Each of these environments has a different cross-section of service requirements that can be specified independently from the others. Each service, however, is defined in a standard form across all environments.

### 2.2 OSE Profile and the APP

An OSE profile is composed of a selected list of open (public), consensus-based standards and specifications that define services in the OSE/RM. Restricting a profile to a specific domain or group of domains that are of interest to an individual organization results in the definition of an organizational profile. The Application Portability Profile (APP) is an OSE profile designed for use by the U.S. Government. It covers a broad range of application software domains of interest to many federal agencies, but it does not include every domain within the U.S. Government’s application inventory. The individual standards and specifications in the APP define data formats, interfaces, protocols, or a mix of these elements.

### 2.3 APP Service Areas

The services defined in the APP tend to fall into seven broad service areas. These service areas are:

- operating system services
- human/computer interface services
- data management services
- data interchange services
- software engineering services
- graphics services
- network services

**Fig. 2:**
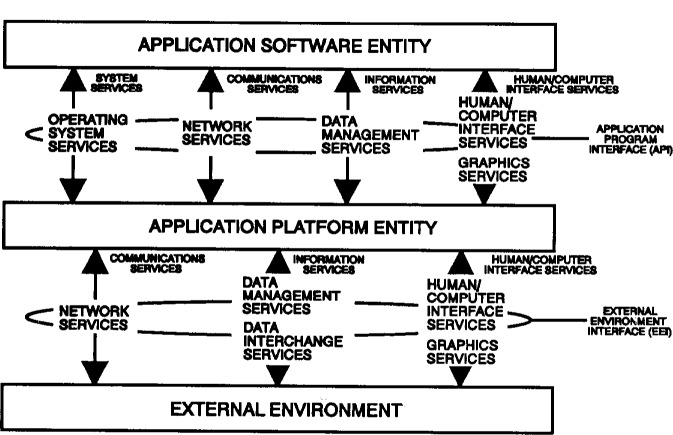
APP service areas and the OSE/RM.

Each service area is defined in the APP. Figure 2 illustrates where each of these seven services areas relates to the OSE/RM. (Assume that software engineering services are applicable in all areas.) Each of the APP service areas addresses specific components around which interface, data format, or protocol specifications have been or will be defined. Security and management services are common to all of the service areas and pervade these areas in one or more forms. Currently, specifications for security can be recommended in operating system services, network services, and access control and integrity constraints for data management services. Specifications for security in the other service areas are not sufficiently advanced to warrant inclusion at this time.

Management services are partly defined and still under development. They are based on the Open System Interconnection (OSI) Network Management Framework, which applies mainly to the overlap among network, system, and application management functions. This overlapping area applies equally to networks and individual nodes on networks and forms the framework for the OSI approach to systems and network management. Other management functions in the typical operating system sense (e.g., user accounts, resource administration, etc.) will be added over time. As these specifications mature and stabilize, they will be reviewed and appropriate ones may be selected for use in the APP.

## 3. Main Program

The main program consisted of a keynote address by David Fisher, standards status by Fritz Schulz, and major issues by NIST staff. The major issues included the Federal Internetworking Requirements Panel, the NIST Virtual Library Program, OSE procurement, and the evolution of the OSE Implementors’ Workshop (OIW).

### 3.1 Keynote Address

Fisher spoke on the Component-Based Software focus area of the Advanced Technology Program (ATP). The ATP’s objective is to promote U.S. economic growth through the development and application of technology. The Component-Based Software focus area defines goals in two areas, business and technical, to meet this objective.

#### Business Goals

Increase Productivity in Software Development by enabling:

- Quality and reliability increases through automation.
- Reduced time to develop and test software.
- Cost amortization from multiple use of software components.

Increase Productivity of Software Users by enabling:

- Increased quality through specialization.
- Improved dependability of systems.
- Enhanced ability to concentrate on core business.

Broaden Addressable Software Markets by enabling:

- Creation of systematically reusable software components.
- Increased interoperation of software.
- Adaptation for use in foreign markets.

#### Technical Goals

Practical Automated Semantic Composition

- Automated composition from independently produced components.
- Interoperation of components in any functionally compatible context.
- Systematic reuse based on semantic characteristics alone.

Automated Tools and Methods for Component-Based Software

- Increase portion of process subject to automation.
- Reduce direct involvement of software experts.
- Technologies with potential for factor of two increases in productivity.
- Enabling tools and processes for component-based software.

Technology to Overcome other Specific Barriers

- Revenue collection on fine-grained software.
- Technology for commercial security needs.
- Interoperation with legacy systems.
- Linguistic mechanisms of incomplete and imprecise specification.
- Robust systems in the presence of errors.
- Improved methods for confirming software dependability.

For additional information on the Component-Based Software focus area of the ATP, contact Dr. Fisher at (301) 975-3649, (301) 926-9524 fax, or dfisher@nist.gov email.

### 3.2 Standards Status

Schulz presented the following updates on the OSE standards activities of IEEE, JTC1, and the Computer Systems Laboratory (CSL) of NIST. The IEEE Portable Operating System Interface (POSIX) Guide is in ballot and is expected to be approved this year. The POSIX guide describes an OSE Reference Model (OSE/RM) that is closely aligned with the APP and that provides a framework for describing open system concepts and defining a lexicon of terms that can be agreed upon generally by all interested parties. The same document is in ballot as a proposed draft technical report (PDTR) within working group (WG)15 of subcommittee (SC)22 of JTC1. The PDTR is also expected to be approved this year. The status of individual programs within the POSIX project were distributed in a handout. Technical Report (TR)10000, OSE profiling, produced by the JTC1 Special Group on Functional Standardization (SGFS) is expected to begin the ballot process this summer. TR 10000 provides a context for functional standardization in support of Open System Environments (OSE). It outlines the basic OSE objectives and concepts, and defines an approach to the taxonomy and format for OSE Profiles specified by International Standardized Profiles. The technical report gives guidance on the nature and content of ISP documents to organizations proposing Draft OSE International Standardized Profiles.

Activities of CSL in the OSE arena reported by Schulz were the new Integration Definition (IDEF) Federal Information Processing Standards (FIPS) and APP planning. Two new FIPS were approved since the last forum. FIPS 183, IDEF0, and FIPS 184 IDEF1X, are excellent tools for system modelling, and are very useful in business process re-engineering. A planned version 3.0 of the NIST APP was announced for calendar year 1995 by Schulz. Participants were very strongly encouraged to provide input to planning meetings that would be held this summer.

### 3.3 Major Issues

The Spring ’94 forum addressed four major issues. These issues were: the Federal Internetworking Requirements Panel (FIRP), the NIST Virtual Library Project, OSE procurement, and the evolution of the OSE Implementors’ Workshop (OIW). Key NIST personnel in each of the above areas made the presentations.

#### 3.3.1 Federal Internetworking Requirements Panel

Tassos Nakassis, Acting Chief, Systems and Network Architecture Division, NIST presented status on the FIRP and the report it has developed. The FIRP was established by NIST to reassess Federal requirements for open systems networks and to recommend policy on the Government’s use of networking standards. The Panel was chartered to recommend actions which the federal government can take to address the short- and long-term issues of interworking and convergence of networking protocols—particularly the Internet Protocol Suite (IPS) and the Open Systems Interconnection (OSI) protocol suite and, when appropriate, proprietary protocols. The draft report from the panel was released in January 1994 and generated about 80 comments despite the short (35 days) comment period. Comments from the United States tended to be supportive, while non-U.S. comments tended to be critical. The panel continues to address comments and plans to publish the report in June 1994. NIST has developed a three-point response to the report. Point one is to modify the Government Open System Interconnection Profile (GOSIP) through an addendum to include Transmission Control Protocol/Internet Protocol (TCP/IP) and related protocols. Initiation of a public debate on the big issues about standards is the second point. This standards debate will seek to answer: Why do they take so long? What is their impact? and What do we need to standardize? The final point is to examine the role of the government in standardization and the actions it needs to undertake in support of the National Information Infrastructure (NII). For further information about the report, contact Nakassis at NIST, Technology Building, Room B217, Gaithersburg, MD 20899.

#### 3.3.2 NIST Virtual Library Program

Paul Vassallo made a presentation on the NIST Virtual Library Program. The program has as its goal bringing to the NIST research and support staffs’ desktop workstations the information, regardless of the form or source, needed to perform their responsibilities. An early step in this program is the Electronic Document Interchange Project, which has as its goal the improvement of the ability of NIST staff to interchange documents across software platforms. The next step is to build on these experiences, developments, and enhancements to improve awareness of and access to the knowledge within the documents. The program has defined document to mean any printable or viewable item. Specific objectives of the program are:

- Universal electronic document exchange.
- Searching and retrieval capability from the desktop.
- Hyperlinked client/server viewing and access.

Vassallo can be contacted at (301) 975-2786, (301) 869-8071 fax, or pvassall@nist.gov email for more information.

#### 3.3.3 OSE Procurement

The Guide on Open System Environment (OSE) Procurements was presented by Gary Fisher. The guidance in the report pertains to U.S. Government acquisition of OSE infrastructure including operating system, human/computer interface, software engineering, data management, data interchange, graphics, network, security, and system/network management services based on implementations of standard application program interfaces, programming languages, data formats, and protocols. Other organizations, such as state and local governments, academic, and private institutions, may also find the information helpful in defining computing environments that promote application portability, interoperability, and scalability. The procurement of information technology that provides an Open System Environment (OSE) can be complex and difficult to manage. Much can be learned from procurement actions that have been instituted for acquiring the technology to support an OSE.

The Open System Environment is spreading throughout applications and systems within the federal government and industry. Agencies are finding that open systems provide a more flexible, cost-effective, and beneficial environment for supporting mission-critical applications than previous infrastructures based on proprietary technology. They are also finding that the initial move to open systems can be expensive and difficult to manage.

Agencies have requested assistance from NIST’s Computer Systems Laboratory (CSL) in an effort to control the evolution of open systems and provide guidance on managing the transition from current environments to the OSE. In the process of implementing open systems, agencies have found that significant up-front planning and current knowledge of technology are necessary to maintain flexibility and to take advantage of targets of opportunity as they arise. Many lessons learned in OSE acquisition programs that agencies have undertaken are included in this report. This information is meant to assist program managers and senior project engineers in acquiring an OSE on which to build flexible, modular systems and applications. For additional information, please contact Gary Fisher at (301) 975-3275, (301) 926-3696 fax, or gfisher@nist.gov email.

#### 3.3.4 OSE Implementors’ Workshop

Ted Landberg, OIW chairman, made the presentation on new work starting in the OIW. The Open Systems Environment Implementors Workshop (OIW) is a public international technical forum for the timely development of implementation agreements based on emerging international standards and public specifications. Its purpose is to broaden the utilization of Open Systems Environment (OSE)-based technologies and to speed their development. The workshop intent is to support the advancement of a technically efficient and compatible technology base for emerging Open Systems on a nationwide basis. The workshop meets four times annually.

The workshop consists of various Working Groups and Special Project Teams. Recently formed working groups are the Multimedia Data & Document Interchange (MDDI) and the Integrated Software Engineering Environments (ISEE). Three other study areas are expected to become working groups in the near future. These study areas are Convergence of TCP/IP and OSI protocols, Electronic Commerce, incorporating Electronic Data Interchange (EDI), and Distributed System Management. For additional information on the OIW, please contact Ted Landberg at (301) 975-2245, (301) 926-3696 fax, or landberg@nist.gov email.

## 4. OSE Users’ Perspective Mini-Workshop

The Users’ Perspective on OSE-based Distributed Systems mini-workshop followed an introductory presentation by Fritz Schulz. Schulz conducted this mini-workshop that presented case studies, lessons learned and plans as they relate to distributed system implementations in government and industry. Speakers from federal agencies and industry discussed their concepts and experiences in planning and implementing distributed systems in their organizations.

### 4.1 Federal Aviation Administration (FAA)

The FAA presentation, “Plans and Strategies for Open Systems,” was made by Roger Cooley, Manager, Corporate Systems Architecture Division (AIT-300), Office of Information Technology. The FAA is organized into a Headquarters, nine regions, and two centers with over 50,000 employees. Their major missions are air traffic control, aviation safety and security, airspace management, airport improvements, inspection, regulation and enforcement. In the past, information technology management and operations were divided into National Airspace Systems (NAS) and non-NAS. The office of Chief Information Officer has been established to integrate these separate activities into one IT organization.

The approach and direction the FAA is taking has many components. Key to the approach is the development of enterprise and inter-enterprise architecture frameworks and the profiles to populate them to support major mission areas and common infrastructure. For each profile a core set of standards will be selected. In the short term this will be a mix of open, consortia, de facto and proprietary standards. In the longer term the target is an open systems, standards-based architecture. To achieve this, the FAA plans to work closely with NIST, standards bodies, and vertical industry groups.

### 4.2 Defense Information Systems Agency (DISA)

John Mitchell, Director, Center for Architecture, Joint Interoperability Engineering Organization, made the presentation for DISA. Architecture definition has been guiding DISA in the area of distributed systems. Their program defines this as “a framework or structure that portrays relationships between or among all elements of a subject force, system or activity.” In practice, an architecture contains a migration path, deficiency analysis, technical insertion, and cost analysis. To support this effort, the Technical Reference Model (TRM) has been developed to specify the services and standards with associated interfaces, protocols, and data formats that support the DOD’s move to an Open System Environment (OSE). There are several critical success factors for distributed systems in the Defense Information Infrastructure (DII). Investment in architecture as a planning mechanism is key. Automation and standardization of the architecture process support efficient use and ensure coordination among architecture programs for interoperability. The resulting architecture is validated with technology insertion projects that build customer and management acceptance.

### 4.3 Boeing Defense and Space Group

J. J. Cinecoe presented the four-point “success enablers” that guide Boeing in their development and use of distributed system. Point one: Involve all vested parties in the development of the architecture. Include managers of all key organizations in the approval of the architecture. The key aspect here is to create special working groups to make the recommendations. Point two: Build a framework to communicate architecture activity. Use the framework over and over and over again. Major elements of the framework are Vision, Principles, Strategy and Standards. Those elements are then cross-referenced in a matrix to the major elements of applications, data, delivery systems and organization. Point three: Plagiarize. There is too much good work and too few really new ideas to justify reinventing the wheel. Sources include: NIST APP, DISA Technical Reference Model and draft OSE/for Immediate Acquisition, Consortia, Standards Bodies, Vendors, and Textbooks. Point four: Advertise. Once you have moved toward open systems, the objective of interoperability will only be achieved if others do the same. Advertise internally, to information systems suppliers, customers, partners, and subcontractors.

### 4.4 Common Issues/Challenges

1. Providing access by the citizens to government data/services via information technology is major challenge with near-term demands.
2. Distributed system architecture process varies considerably across organizations. Consensus is the common view of the technology. Any formulation of process must be flexible.
3. Coupling distributed system architecture with development and procurement is critical for success.
4. A strong benefit of OSE-based distributed system architecture process is ease of planning for technology insertion.
5. Distributed system architecture process is a team discipline due to the breadth of skills and consensus required.

## 5. Distributed System Management Mini-Workshop

An introductory presentation to the Distributed System Management mini-workshop was made by Joe Hungate. Hungate then conducted the mini-workshop. A presentation on requirements for operation and administration of OSE-based distributed systems kicked off the session. The presentation summarized the start of a new work item to augment the APP in the management service area. Objectives of the program are integrated approach, consistent user interface, interoperability among diverse resources, based on operational context, and flexibility to allow operation in different domain types and relationships.

Major domain areas within the operation and administration of OSE-based distributed systems include system administration, network management, security management and information management. Collectively, these domains constitute the functionality required to operate and administer a distributed system. Activities in the operation and administration of OSE-based distributed systems can take on a very broad scope. This scope can be as simple as a single open system taking operation and administration responsibility for one or more dependent systems, or as complex as those in which there is a transient negotiated division of operation and administration activities. The scope can vary infinitely between the two stated above. The prescribed manner by which these distributed systems or constituent components interact determines their relationship. Major categories of domain relationships are: centralized, federated, or hierarchical.

Paul Brusil was the second speaker and presented information on network management. Network management is based on ISO/ITU-T^1^ System Management Technology. The ISO approach to management consists of suites of standards. The four suites are basic structure standards (framework), system management function standards, system management communication standards and system management information standards. Utilizing the definition of managed objects these suites can manage a varying collection of OSI nodes. Future work is planned to expand management to non-OSI nodes.

A list of requirements for the operation and administration of OSE-based distributed systems was presented for open discussion. The list of requirements and an issues list that was developed by the mini-workshop follows:

System Administration Requirements

- Application Software Management
- Application Platform Management
- Accounting Management
- Backup and Restore Management
- Configuration Management
- File System Management
- Device Management
- Fault Management
- License Management
- Performance Management
- Policy Management
- Print Management
- Software Installation and Distribution
- System Startup and Shutdown
- User and Group Management

Information Management Requirements

- Data Administration
- Database Management

Security Management Requirements

- Identification/Authentication
- Access Control
- Data and System Integrity
- Audit
- Confidentiality
- Non-repudiation

### Issues List

1. Establish common definitions and terms. Do not use the term ensemble. It is potentially confusing and provides no value beyond use of the term Profile.
2. Establish liaisons with Data Management Technical Panel (DMTP), former Unix International (UI),….
3. Address metrics as a separate work area.
4. Identify requirements, resources.
5. Feedback findings to the APP.
6. Encourage Vendor participation.
7. Utilize lessons learned from users’ operational experience.
8. Provide for international interoperability.
9. Identify framework/reference model to guide work.

The distributed system management working group will begin meeting as a subcommittee of the OSE-TC within the OIW structure. For further information please contact Joe Hungate at: (301) 975-3368, (301) 926-3696 fax, or jhungate@nist.gov email.

## Footnotes

1 ITU-T was formerly CCITT.
